# Obstructive uropathy in a female infant with a single kidney: Unmasking congenital urethral stenosis: A case report and review of the literature

**DOI:** 10.1016/j.ijscr.2025.112016

**Published:** 2025-10-10

**Authors:** Yasmine Houas, Hela Oueslati, Nour Ben Alaya, Nader Bennour Ghaddab, Riadh Jouini

**Affiliations:** Pediatric Surgery Department “A”, Children Hospital Bechir Hamza, Tunis El Manar University, Tunisia

**Keywords:** Congenital urethral stenosis, Vesicoureteral reflux, Solitary kidney, Pediatric urology, Bladder outlet obstruction, Vesicostomy

## Abstract

**Introduction and importance:**

Congenital urethral stenosis (CUS) in female infants is an exceptionally rare urological anomaly. When combined with vesicoureteral reflux (VUR) and a solitary functional kidney, it presents a significant risk for renal deterioration. Early recognition is essential to avoid irreversible damage, especially in complex cases with multiple comorbidities.

**Case presentation:**

We report the case of a 33-month-old female born prematurely at 26 weeks, with a history of omphalocele repair, ventriculitis, retinopathy of prematurity, and a chromosomal abnormality. She presented with recurrent febrile urinary tract infections and worsening hydronephrosis of her only functional kidney. Multiple catheterization attempts failed due to a pinhole-sized urethral meatus. Examination under anesthesia revealed congenital urethral stenosis, which was managed with serial dilations allowing catheter placement and voiding cystourethrogram (VCUG). Imaging showed a trabeculated bladder with diverticula, grade V VUR, and laterally displaced ureteral orifice. Due to persistent infections and poor compliance with catheterization, a vesicostomy was performed. The patient subsequently remained infection-free, with improved renal function and resolution of hydronephrosis.

**Clinical discussion:**

This case highlights the diagnostic challenge posed by CUS in females, particularly in the context of solitary kidney and developmental delay. The absence of obvious obstructive symptoms may delay diagnosis. In such complex scenarios, vesicostomy provides effective bladder drainage, protects upper tract function, and simplifies care when clean intermittent catheterization is not feasible.

**Conclusion:**

CUS should be included in the differential diagnosis of bladder outlet obstruction in female infants, particularly those with recurrent UTIs and solitary kidney. In carefully selected patients, vesicostomy remains a valuable interim or long-term solution to preserve renal function and improve quality of life**.**

## Highlights

• Congenital urethral stenosis (CUS) in females is rare yet significant, often causing obstruction and recurrent UTIs.
• Symptoms include weak stream, straining, dribbling, enuresis, and recurrent UTIs, often misdiagnosed as functional disorders.
• Severe CUS may cause VUR, hydronephrosis, or renal failure, particularly in patients with a single kidney.
• Diagnosis is challenging, often requiring exam under anesthesia, urethroscopy, calibration, and imaging such as VCUG.
• Treatment includes dilation, meatotomy, and for refractory cases, urethrotomy or meatoplasty. Outcomes vary with restenosis risk.

## 1 Introduction

Congenital anomalies of the female urethra are rare and frequently overlooked in clinical practice due to their subtle presentation. Unlike in male infants—where congenital urethral stenosis (CUS) is commonly recognized as part of the posterior urethral valve spectrum—this condition is scarcely reported in females [1]. Its rarity, combined with the nonspecific nature of symptoms such as recurrent urinary tract infections (UTIs), weak urinary stream, or voiding dysfunction, often leads to misdiagnosis or delayed recognition [2]. When unrecognized, CUS can result in progressive bladder dysfunction and irreversible upper urinary tract damage, especially in patients with a solitary functioning kidney, where renal reserve is already limited.

We present this case to highlight the diagnostic challenges and serious consequences of untreated CUS in female patients. The unique combination of a functional solitary kidney, neurodevelopmental delay, and recurrent UTIs underscores the need for a high index of suspicion when evaluating voiding dysfunction in young girls. The staged therapeutic approach—combining vesicostomy with serial urethral dilations—resulted in favorable renal outcomes. This case emphasizes the importance of early urological evaluation and intervention, particularly in high-risk pediatric populations, and aims to raise awareness of an often-overlooked diagnosis. A brief review of the literature is provided to contextualize the clinical decision-making and underline the rarity of this condition. This case report has been reported in line with the SCARE checklist [3].

## 2 Case presentation

We report the case of a 33-month-old girl born prematurely at 26 weeks and 3 days of gestation, who was referred for recurrent febrile urinary tract infections (UTIs) and worsening hydronephrosis of a solitary functioning kidney. Her medical history was notable for a complex neonatal course. She experienced perinatal asphyxia necessitating prolonged intubation and mechanical ventilation, and underwent surgical repair of an omphalocele on day 6 of life. Her early postnatal period was further complicated by ventriculitis, resulting in psychomotor developmental delay. Additional comorbidities included bilateral retinopathy of prematurity and cerebral atrophy, as confirmed by neuroimaging. Genetic analysis revealed a structural abnormality of chromosome 14, though without a specific syndromic correlation.

Since infancy, she was followed for left-sided hydronephrosis and a non-functioning right kidney, consistent with a congenital solitary left kidney, as the right kidney was never visualized on serial ultrasonography.

**Fig. 1:**
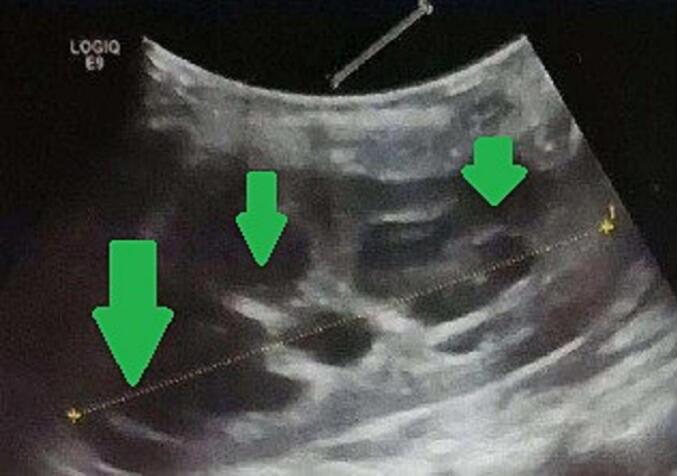
Renal ultrasound showing urinary dilation of the left kidney (Arrow).

**Fig. 2:**
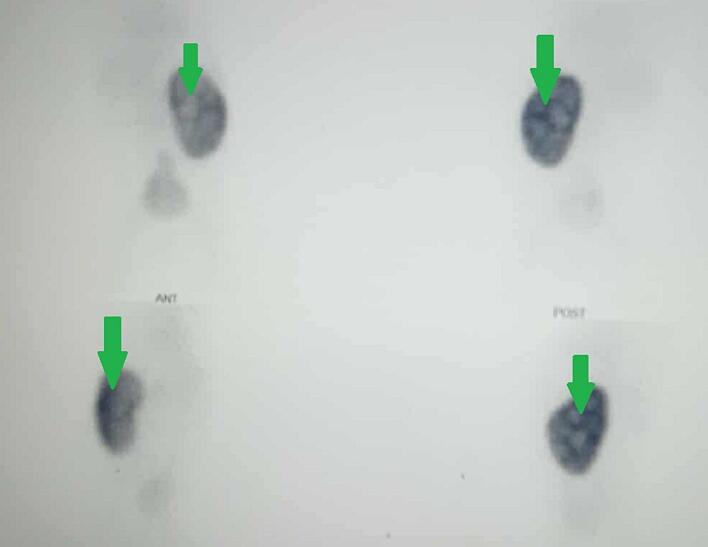
DMSA scan showing absence of uptake in the right kidney and heterogeneous uptake in the left kidney (Arrow).

At 5 months of age, renal ultrasound showed left hydronephrosis with a thin-walled bladder and a diverticula of 7 mm (Fig. 1). Later DMSA scans demonstrated a solitary left kidney with preserved contours but heterogeneous tracer uptake and hypofixation areas, likely related to collecting system dilatation. No renal scarring was observed (Fig. 2). Over time, however, repeated ultrasounds and DMSA imaging revealed progressive cortical thinning and worsening hydronephrosis, suggesting obstructive uropathy.

Biochemical monitoring showed persistently elevated serum creatinine, reaching 51 μmol/l at 33 months. According to the Schwartz formula, her estimated creatinine clearance was 59.5 ml/min/1.73 m^2^. Blood pressure remained within normal limits, and urinalysis revealed no albuminuria. Antibiotic prophylaxis was initiated early in the course of recurrent UTIs.

**Fig. 3:**
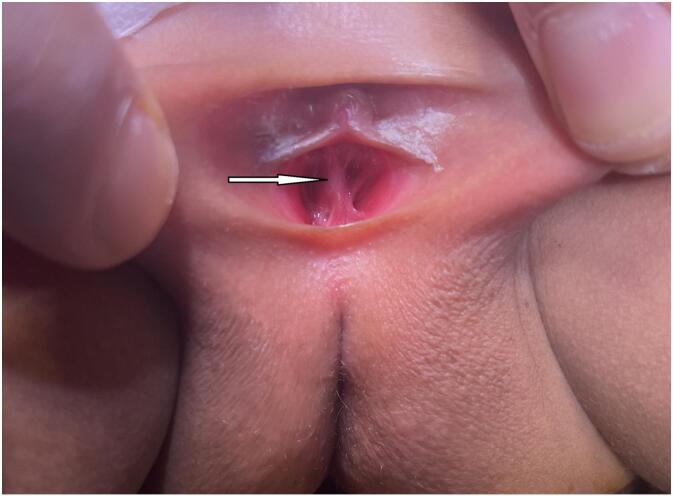
Physical examination showing a punctiform urethral orifice (Arrow).

Despite multiple attempts, urethral catheterization was repeatedly unsuccessful due to extreme narrowing of the urethral meatus, which appeared as a pinhole orifice on physical examination (Fig. 3). An examination under anesthesia (EUA) was therefore performed, revealing a severely stenotic urethral orifice that barely admitted the tip of a fine probe.

**Fig. 4:**
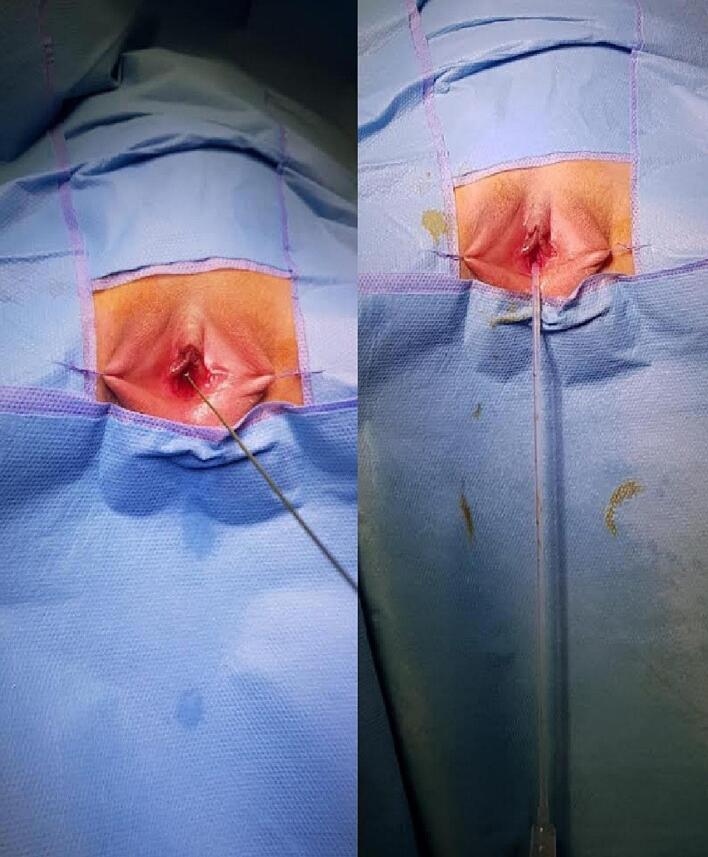
Gentle dilations of the urethra eventually allowed passage of a 6 French catheter.

**Fig. 5:**
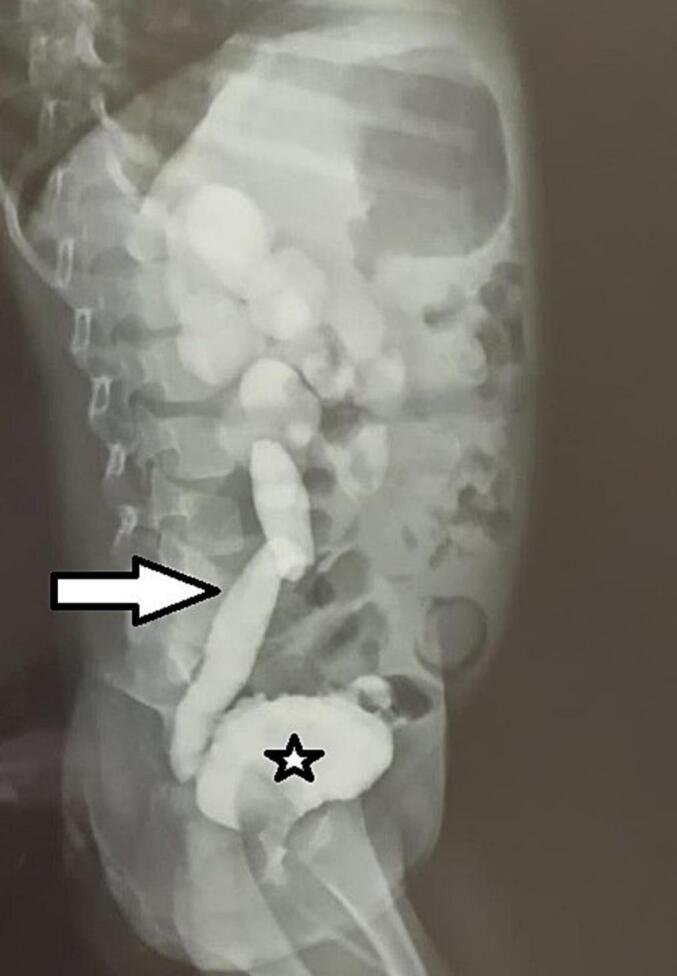
The VCUG showing a thick-walled, trabeculated bladder with multiple diverticula (Star) and severe grade V vesicoureteral reflux (Arrow).

Endoscopic access was initially impossible due to a distal obstructing membrane consistent with congenital urethral stenosis (CUS). Serial gentle dilations of the urethra eventually allowed passage of a 6 French catheter (Fig. 4), enabling a voiding cystourethrogram (VCUG). The VCUG demonstrated a thick-walled, trabeculated bladder with multiple diverticula, severe grade V vesicoureteral reflux (VUR) on the left side (Fig. 5).

Given the patient's neurodevelopmental delay and poor tolerance of intermittent catheterization, a vesicostomy was performed to ensure continuous bladder drainage and reduce infection risk.

Postoperative follow-up was scheduled every 3 months, including clinical examination, renal and bladder ultrasound, and laboratory tests such as serum creatinine and estimated glomerular filtration rate (eGFR). Annual DMSA scans were planned.

During follow-up, the patient showed clear clinical and radiological improvement. She remained free of UTIs, her serum creatinine progressively decreased, and imaging confirmed regression of hydronephrosis, and reduced ureteral dilatation.Given the patient's neurological state, she is maintained with a vesicostomy.

## 3 Discussion

Congenital urethral stenosis (CUS) in females is an exceedingly rare but clinically significant condition. Historically, its recognition dates back to Barthélémy-Cabrol in 1552, who described distal meatal obstruction in a girl, and subsequent authors confirmed the existence of various forms of distal urethral stenoses in females, notably complete, incomplete, and intraluminal strictures within the distal third of the urethra [[1], [2], [3], [4]].

From an anatomical and embryological perspective, these stenoses likely arise from incomplete canalization of the urogenital sinus or the persistence of embryologic membranes [5,6]. Pathologically, congenital stenosis is initially a simple narrowing without inflammation, but repeated infections often result in periurethral fibrosis, exacerbating the obstruction and perpetuating a cycle of infection and scarring [7,8].

The clinical presentation of CUS is variable but often includes obstructive voiding symptoms such as a weak urinary stream, straining, dribbling, and recurrent UTIs. In many cases, symptoms manifest during infancy or early childhood and may lead to misdiagnoses such as enuresis or dysfunctional voiding [7,8].

Although early pediatric series suggested that distal stenosis may be more common than reported [3,4], the absence of standardized criteria continues to hinder accurate diagnosis [9].

Physical findings are often subtle, and imaging alone is insufficient. VCUG may reveal distal narrowing, bladder trabeculation, diverticula, or vesicoureteral reflux, but such changes can also occur in functional or neurogenic bladder disorders, limiting specificity [9]. Examination under anesthesia with careful calibration and cystoscopy remains the most reliable method to confirm a fixed obstruction and to assess secondary bladder changes [6,7]. In our case, the inability to catheterize the patient prompted examination under anesthesia, which remains a key step in confirming the diagnosis while avoiding trauma in uncooperative children [8].

Urodynamic testing may help exclude functional voiding disorders but cannot independently establish the diagnosis of CUS [10].

.CUS must be differentiated from neurogenic bladder which may also present with trabeculation, incomplete emptying, and recurrent UTIs. In CUS, a rigid distal ring can be identified endoscopically or with calibration, whereas in neurogenic bladder the dysfunction is diffuse without a discrete narrowing [6].

Treatment of female urethral stenosis has evolved over time. Historically, dilation and meatotomy have been the primary interventions. While dilation is often sufficient and provides rapid symptom relief, it may not be curative in all cases due to the risk of restenosis [1,7]. Periodic dilation has shown varying success rates, with some authors advocating it as a first-line treatment, especially when combined with antibiotic prophylaxis in patients with recurrent UTI [11,12]. However, other studies have questioned the long-term efficacy of dilation and emphasized the need for more definitive interventions such as meatoplasty or urethrotomy in refractory cases [1,13,14].

Table 1 summarizes reported cases of female congenital urethral stenosis, including presenting symptoms, diagnostic methods, associated vesicoureteral reflux (VUR), stenosis type, treatment approaches, and clinical outcomes.

**Table 1:** Literature review.

Title (Reference)	Author (Year)	Country	Sample size (Median Age)	Symptoms^a^	Diagnosis	Associated VUR	Type of urethral stenosis	Treatment	Outcomes
Congenital obstruction of female urethra [8]	Addison (1932)	UK	1 (4 yrs)	Chronic UTI, dribbling, bladder distension	VCUG, EUA	Yes (bilateral hydronephrosis)	Mid-urethral diaphragm	Dilation, catheterization	Death due to renal failure
Congenital obstructions of the female urethra [3]	Stevens (1936)	USA	5 (1–8 yrs)	Dysuria, recurrent UTI, emaciation	Cystoscopy	Yes (bilateral in some cases)	Meatal or internal urethral stricture	Dilation	Symptom resolution, some required repeat dilation
Urethral Stenosis in Young Girls [7]	Schulte & Williams (1952)	USA	7 cases (1–11 yrs)	Fever, UTIs, dysuria, enuresis	EUA, cystoscopy	Few with VUR	Mostly meatal	Dilation	Most cases symptom-free after dilation
Distal urethral obstruction in children [9]	Shopfner (1967)	USA	478 children (287 girls)	Recurrent UTIs	VCUG, calibration	Some cases	Mostly meatal or distal urethral stenosis	Dilation	Symptom improvement in selected cases
Congenital urethral valve in female [15]	Mitchell et al. (1967)	USA	1 (9.5 months)	Vomiting, fever, pyuria	VCUG, EUA	Yes (right-sided)	Urethral valve	Endoscopic ablation	Upper tract decompression
Significance of distal urethral stenosis in young girls: experience with 241 cases [16]	Brannan et al. (1969)	USA	241 girls	Enuresis, fever, dysuria	Cystoscopy, dilation	Yes (confirmed in 46)	Distal urethral narrowing	Meatotomy	Good infection control, positive cultures in 107 pts
Distal urethral stenosis in female [17]	Wyatt (1975)	Canada	72 girls (<16 yrs)	Enuresis, frequency, recurrent UTI	VCUG, endoscopy	Yes (approx. 40 %)	Distal urethral ring, meatal	Dilation, urethrotomy	>80 % symptomatic relief
Renal scarring in vesicoureteric reflux and distal urethral stenosis treated by urethrotomy [18]	Andresen et al. (1982)	Denmark	34 (Mean 7 yrs)	UTI, radiologic signs of reflux	VCUG, IVP	Yes (grades 1–3)	Distal urethral stenosis	Otis urethrotomy	Improved infection rate; 21 % still had UTI
urethral meatal stenosis causing severe hydronephrosis [19]	Bueschen & Royal (1986)	USA	1 (2 months)	Fever, bilateral hydroureteronephrosis	IVP, VCUG, cystoscopy	No	Meatal stenosis	Dilation	Improved renal function, good stream post-op
Surgical therapy of distal urethral stenoses [1]	Biewald & Duda (1987)	Germany	163 (Mean age: 7.5 yrs)	Recurrent UTIs	Calibration, EUA	35.1 % pre-op	Meatal (incomplete/complete), distal strict sense	Meatoplasty	83.5 % cured; 25.5 % VUR improved
Stenosis of external urethral opening in girls [10]	Milanović et al. (1989)	Serbia	105 (Mean age: 8.4 yrs)	Recurrent UTIs, dysuria	Clinical, EUA	36.8 % had VUR (grades I–II)	Distal meatal stenosis	Meatoplasty	78.1 % infection resolution; partial VUR improvement
Two cases of congenital urethral stenosis with VUR [14]	Watanabe & Omatsu (1990)	Japan	2 (3 & 7 yrs)	UTI	VCUG, EUA	Yes	Anterior (boy), peripheral (girl)	Urethroplasty, dilation	VUR resolved within 1 year
Congenital stenosis of external orifice of urethra in female [20]	Zhengzhou Shi et al. (2017)	China	1 (5 yrs)	Thin urine stream, intermittent UTIs	VCUG, EUA	Yes (grade III)	Meatal stenosis	Meatoplasty	No recurrence at 1-year follow-up
Congenital meatal urethral stenosis [21]	Arredondo Montero et al. (2023)	Spain	1 (6 yrs)	Altered urine stream	Uroflowmetry, EUA	No	Meatal stenosis with membrane	Ventral meatotomy	Symptom resolution
Obstructing urethral membrane [22]	Jariwala et al. (2024)	India	1 (Toddler)	Straining, UTIs	EUA, cystoscopy	Not specified	Obstructing membrane	Endoscopic ablation	Clinical resolution

a UTI: urinary tract infection, VCUG: voiding cystourethrogram, EUA: examination under anesthesia, VUR: vesico urethral reflux, IVP: intravenous pyelogram.

In our patient, dilation enabled relief and allowed for VCUG to be performed, confirming high-grade VUR and a diverticular bladder. Given the patient's neurodevelopmental challenges and the impracticality of intermittent catheterization, a vesicostomy was performed as a temporizing measure. This intervention ensures low-pressure drainage, reduces infection risk, and protects the solitary kidney while preparing for definitive reconstruction [6,14].

Prognostically, the most critical concern in CUS is its impact on renal function, especially in children with a solitary kidney. Prolonged bladder outlet obstruction can elevate intravesical pressure, compromising the anti-reflux mechanism and leading to secondary VUR and hydronephrosis [15,23]. Early diagnosis and timely surgical intervention are essential to halt the cascade of renal deterioration, as untreated cases can lead to irreversible renal damage and uremia [7,9].

## 4 Conclusion

This case highlights the importance of considering congenital urethral stenosis in female infants with recurrent urinary tract infections and obstructive uropathy, particularly when a solitary functional kidney is involved. Reporting such rare presentations is essential to raise clinical awareness, reduce diagnostic delay, and guide timely intervention. In complex patients with comorbidities, early vesicostomy can be a valuable, kidney-sparing option that stabilizes the urinary tract.

## Consent

Written informed consent was obtained from the patient's parents/legal guardian for publication and any accompanying images. A copy of the written consent is available for review by the Editor-in-Chief of this journal on request.

## Ethical approval

Study is exempt from ethnical approval in the institution of the authors.

## Author contributions

Y.H. conceived the project, developed the software, and drafted the manuscript. H.O. and N.E.H.B.A. contributed to content validation, data analysis, and manuscript revision. N.B.G. and R.J. supervised the work, validated the clinical aspects, and approved the final version. Y. H is the guarantor of the work.

## Sources of funding

No funding was obtained for this research.

## Declaration of competing interest

The authors declare no conflicts of interest regarding this manuscript.
